# A Miracle That Accelerates Operating Room Functionality: Sugammadex

**DOI:** 10.1155/2014/945310

**Published:** 2014-08-14

**Authors:** Erdal Dogan, Mehmet Salim Akdemir, Abdulmenap Guzel, Mehmet Besir Yildirim, Zeynep Baysal Yildirim, Mahir Kuyumcu, Abdurrahman Gümüş, Hakan Akelma

**Affiliations:** ^1^Department of Anesthesiology and Reanimation, Faculty of Medicine, Dicle University Medical School, Diyarbakir, Turkey; ^2^The Education and İnvestigation Hospital of Diyarbakir, Turkey; ^3^The Pediatrics Hospital of Diyarbakir, Turkey; ^4^Tatvan State Hospital, Bitlis, Turkey

## Abstract

*Background.* Sugammadex offers a good alternative to the conventional decurarisation process currently performed with cholinesterase inhibitors. Sugammadex, which was developed specifically for the aminosteroid-structured rocuronium and vecuronium neuromuscular blockers, is a modified cyclodextrin made up of 8 glucose monomers arranged in a cylindrical shape. *Methods.* In this study, the goal was to investigate the efficacy of sugammadex. Sugammadex was used when there was insufficient decurarisation following neostigmine. This study was performed on 14 patients who experienced insufficient decurarisation (TOF <0.9) with neostigmine after general anaesthesia in the operating rooms of a university and a state hospital between June, 2012, and January, 2014. A dose of 2 mg/kg of sugammadex was administered. *Results.* Time elapsed until sugammadex administration following neostigmine 37 ± 6 min, following sugammadex it took 2.1 ± 0.9 min to reach TOF ≥0.9, and the extubation time was 3.2 ± 1.4 min. No statistically significant differences were detected in the hemodynamic parameters before and after sugammadex application. From the time of administration of sugammadex to the second postoperative hour, no side effects or complications occurred. None of the patients experienced acute respiratory failure or residual block during this time period. *Conclusion.* Sugammadex was successfully used to reverse rocuronium-induced neuromuscular block in patients where neostigmine was insufficient.

## 1. Introduction

In addition to their role in successful endotracheal intubations, muscle relaxants are also important in making surgical interventions safer, more comfortable, and quicker [1]. Postoperative residual curarisation following muscle relaxant use is defined as the presence of nicotinic receptors that remain blocked in postoperative patients. Even in cases where no symptoms are present, 60–70% of receptors may remain curarised postoperatively [2]. The cholinesterase inhibitor agents used for conventional decurarisation have many adverse effects. Due to the lack of nicotinic selectivity with these agents, many serious side effects can occur due to the stimulation of the muscarinic nervous system. Examples of side effects include bradycardia, QT prolongation, bronchoconstriction, hypersalivation, and hypermotility. In order to avoid these side effects, decurarisasation is performed, generally by coadministering an anticholinergic agent (atropine, glycopyrrolate, etc.) [3].

Sugammadex is a current alternative to the conventional decurarisation traditionally performed with cholinesterase inhibitors. Sugammadex is a modified cyclodextrin that was engineered to reverse the effects of aminosteroid muscle relaxants, modified further for optimal affinity rocuronium [4]. Cyclodextrin made up of 8 glucose monomers arranged in a cylindrical shape. A sugammadex molecule noncovalently binds rocuronium or vecuronium molecules in the plasma, thus causing a decrease in the plasma concentrations of these agents. A gradient is formed that allows rocuronium/vecuronium to pass from the extravascular space into towards the blood. Thus, fast elimination and decurarisation are achieved. When decurarisation is performed via this mechanism, recurarisation and muscarinic side effects are not observed [2].

In this study, the goal was to investigate the efficacy of sugammadex for use during insufficient decurarisation following neostigmine.

## 2. Materials and Methods

In this study, we retrospectively analyzed data from 14 patients who received sugammadex due to insufficient decurarisation (TOF < 0.9) following neostigmine administration for postoperative reversal of the effects of neuromuscular blocking agents in the operating rooms of a university and a state hospital between June, 2012, and January, 2014. Patients with liver and renal failure, pregnant women, those who experienced postoperative acidosis as determined by arterial blood gas, hypothermia, muscle disease, or those with known allergies to the drugs used were not included in the study.

Prior to surgery, patients were attached to monitors which measured the ECG, SpO_2_, and noninvasive arterial blood pressure in addition to routine monitoring, along with an accelomyography device (TOF Watch SX) set to stimulate the ulnar nerve in order to evaluate the neuromuscular block. Train of four (TOF) electrodes was fixed to the ulnar edge of the distal forearm, a temperature probe was placed on the palm, and the transducer was put on the inner side of the thumb. The hand and forearm were wrapped in cotton to prevent the peripheral temperature from dropping below 32°C.

Following the induction of anaesthesia, 0.6 mg/kg of rocuronium was administered to the patients as a muscle relaxant. The TOF device was set to take a measurement every 15 seconds. The patients were intubated when the TOF was zero, and anaesthesia was maintained with 50% O_2_ + N_2_O and 1% sevoflurane.

The patients' heart rate (HR), mean arterial pressure, and SpO_2_ values were recorded during surgery, right before sugammadex administration, and at the first and fifth minutes following sugammadex administration.

Intravenous fentanyl was used as an analgesic during surgery. At the end of the operation, low concentration sevoflurane and N_2_O administration were stopped, and 100% O_2_ was started. When respiratory movements were observed, antagonization was performed with 15 mcg/kg of atropine and 35 mcg/kg of neostigmine. An additional 15 mcg/kg of neostigmine was administered to patients who did not spontaneously breath or who had low tidal volumes, tachycardia, and tachypnea after antagonization despite spontaneous breathing. In patients with continuing tachycardia and tachypnea, spontaneous breathing was supported with 100% O_2_. A 2 mg/kg IV bolus of sugammadex was administered to patients who still could not open their eyes, swallow, raise their head, and had TOF values < 0.9 after neostigmine administration. The patients were extubated following sugammadex injection once the TOF value was ≥0.9. After extubation, the patients were monitored for acute respiratory failure, residual block, and any side effects of sugammadex for up to 2 hours postoperatively.

Patients' demographic data, duration of surgery, hemodynamic parameters, the muscle relaxant agent used, time elapsed until sugammadex administration following neostigmine, duration of time to reach TOF ≥ 0.9, extubation time, Aldrete score at the fifth after extubation minute, and any side effects or complications up to 2 hours after surgery were recorded.

Statistical analyses were performed using the SPSS 16.0 for Windows (SPSS Inc, Chicago, IL, USA) program. Descriptive statistics of the demographic data and continuous variables were expressed as mean ± standard deviation. Nonparametric, dependent data were evaluated with a Wilcoxon test. A* P* value of <0.05 was considered statistically significant.

## 3. Results

The patient demographic data is shown in Table 1. Three patients were ASA II, 8 patients ASA III, and 3 patients ASA IV.

**Figure 1:**
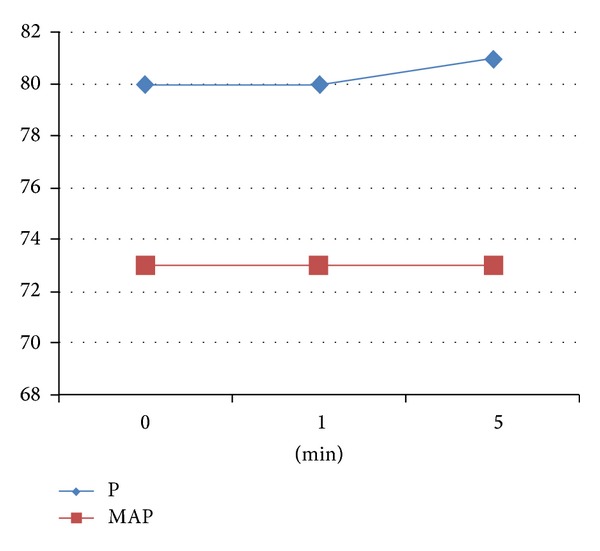
Mean pulse rate and blood pressure values before sugammadex administration (0 min) and at the first and fifth min following sugammadex administration (p: pulse/min, map: mean arterial pressure/mmHg).

Immediately before sugammadex administration, the patients had a mean pulse rate of 80 beats/min and mean arterial pressure (MAP) of 73 mmHg. The patients' pulse rate at the first minute following sugammadex administration was 80 beats/min and their mean arterial pressure (MAP) was 73 mmHg. At the fifth minute following sugammadex administration, the mean pulse was 81 beats/min and the mean arterial pressure was 73 mmHg. The patients' pulse rate before and 1 minute and 5 minutes after sugammadex administration did not differ significantly (*P* > 0.05, *P* > 0.05). The mean arterial pressure of the patients before and 1minute and 5 minutes after sugammadex administration also did not differ significantly (*P* > 0.05, *P* > 0.05 (Figure 1)).

The time to reach TOF ≥ 0.9 was 2.1 ± 0.9 min, and the extubation time was 3.2 ± 1.4 min following sugammadex administration to the patients included in the study (Table 2).

No side effects or complications were encountered in patients from the time of sugammadex administration to the second postoperative hour. None of the patients exhibited acute respiratory failure and residual block during this time period.

## 4. Discussion

In this study, we investigated the use of sugammadex for cases in which neostigmine was insufficient to reverse the effects of neuromuscular blocking agents (TOF < 0.9).

A residual neuromuscular block refers to postoperative symptoms and signs of muscle weakness due to incomplete reversal of the effects of nondepolarizing neuromuscular blockers used intraoperatively. Objective monitoring methods can be used during general anesthesia to evaluate neuromuscular transmission, such as accelomyography and mecanomyograph. These monitoring methods have revealed that the residual curarisation incidence due to nondepolarizing neuromuscular blockers is fairly high [5].

There are differences in the various anticholinesterase drugs used to antagonize the residual effects of nondepolarizing muscle relaxants. In the US, reversal therapy after anaesthesia is performed routinely, whereas, in some European countries, it is not common practice. In our clinic, neostigmine and atropine are routinely used to reverse the effects of nondepolarizing neuromuscular agents.

Reservations regarding the reversal of a competitive neuromuscular block with anticholinesterase agents stem from the possible side effects. In addition to the potential cardiovascular effects of atropine, glycopyrrolate, neostigmine, and edrophonium, neostigmine causes dry mouth, nausea, vomiting, bronchospasm, and increased bowel movements [6].

In recent years, sugammadex, which is a specific cyclodextrin, has been developed to antagonize the neuromuscular block caused by steroidal neuromuscular blocking agents such as rocuronium and vecuronium [7–9]. Sugammadex works by quickly reducing the number of rocuronium molecules on the nicotinic receptors of the motor endplate by the rocuronium molecules in the plasma. Thus, it allows muscle activity to resume in a short period of time. Neostigmine is not a direct pharmacological antagonist of NMBAs. Rather, neostigmine inhibits acetylcholine esterase. The accumulated acetylcholine then competitively displaces the NMBA from the receptor. This is only effective when the NMBA concentration in the neuromuscular junction has already decreased, that is, not at a profound deep neuromuscular block. In contrast, suggamadex chelates all the free NMBA in the plasma quickly, thereby creating a large concentration gradient between the neuromuscular junction and the plasma for free NMBA. The resulting diffusion of NMBA from neuromuscular junction to plasma causes a drop in end plate occupancy of NMBA which occurs much more quickly than after reversal by neostigmine [8, 10].

In a study conducted by Shields et al., the ability of different doses of sugammadex to eliminate the neuromuscular block induced by rocuronium was compared [11]. Thirty patients were initially given 0.6 mg/kg of rocuronium, causing a deep block for at least two hours. When a TOF of 2 was reached, 0.5 mg/kg, 1 mg/kg, 2 mg/kg, 4 mg/kg, and 6 mg/kg of sugammadex were administered. The time it took to reach a TOF of 0.9 was found to be 6.4, 2.4, 2.3, 1.4, and 1.2 min, respectively.

Suy et al. conducted a similar study, in which 0.6 mg/kg of rocuronium was administered as the neuromuscular blocker. Sugammadex was administered after reappearance of the second muscle twitch, and the time it took to reach a TOF of 0.9 was found to be 31.8, 3.7, and 1.1 min, following placebo, 0.5 mg/kg, and 4 mg/kg of sugammadex, respectively [12].

In our study, it took 2.1 ± 0.9 min to reach a TOF of 0.9 after 2 mg/kg sugammadex administration, which is consistent with the previous studies.

Lenz et al. reported a case of acute respiratory failure due to residual neuromuscular block in a patient with chronic renal failure, in which 5 mg neostigmine and 1 mg glycopyrrolate were insufficient to fully antagonize the neuromuscular block created by 10 mg of vecuronium during induction of anesthesia [13]. Following the administration of 350 mg sugammadex (4 mg/kg), in approximately 60 seconds, tidal volumes were restored to normal values and the patient was extubated after 2 minutes.

Tuzcu et al. described a male patient with a suspicious spinal mass who was difficult to intubate even after the administration of 100 mg succinylcholine, a 50 mg loading dose of rocuronium, and a 5 *μ*g/kg/min infusion used to maintain muscle relaxation [14]. Despite the administration of 50 *μ*g/kg of neostigmine and 15 *μ*g/kg of atropine, at the end of the surgery the patient had spontaneous respiration but still had low tidal volumes, tachycardia, and tachypnea and clinically was not improving. An IV bolus of 200 mg of sugammadex (about 2 mg/kg) was injected 45 minutes later. Thirty seconds after the injection, the patient's tidal volumes rose above 450 mL and his inspiratory strength reached −25 cm H_2_O and he was extubated within 90 seconds.

de Menezes et al. described the use of 1.2 mg/kg of rocuronium as a neuromuscular blocker during induction of anaesthesia in a 65-year-old patient who received emergency surgery due to an acute abdomen [15]. The surgical procedure was completed 90 min after the induction of anaesthesia. The patient had received 50 *μ*g/kg neostigmine and 30 *μ*g/kg atropine for decurarisation and 3 minutes later exhibited a T4/T1 ratio of under 0.3. After waiting for 45 min following neostigmine, the patient still lacked sufficient muscle strength for safe extubation and had a T4/T1 ratio below 0.4. Thus, 2 mg/kg of sugammadex was administered, and the patient was extubated within 2 min once his T4/T1 ratio rose above 0.9. No residual neuromuscular block findings were observed in the patient who was monitored postoperatively for 2 hours.

The above studies describe the successful use of sugammadex in cases in which neostigmine was insufficient for the reversal of a rocuronium-induced neuromuscular block. Our study is consistent with the literature. In our study, we did not observe any side effects after sugammadex use.

Because sugammadex is costly, it still has not been routinely used. However, given its ability to quickly and effectively reverse a deep neuromuscular block, low incidence of side effects, and lack of known serious drug interactions, it is expected to be routinely used in the near future in order to accelerate operating room functionality. There is a need for studies on a larger series of patients.

## Figures and Tables

**Table 1:** Demographic data.

Gender (M/F)	4/10
Age (years) ± SD	62 ± 14
Height (cm) ± SD	165 ± 5
Weight (kg) ± SD	76 ± 18
Surgery time (min) ± SD	174 ± 88

**Table 2:** Time elapsed between neostigmine and sugammadex administration, time to reach TOF ≥ 0.9, extubation duration, and Aldrete score.

Time elapsed until sugammadex administration following neostigmine	37 ± 6 min
Time to reach TOF ≥ 0.9	2.1 ± 0.9 min
Extubation time	3.2 ± 1.4 min
Aldrete score	9
